# Functional connectivity of youth in family-like residential care in Japan: Impact of reactive attachment disorder and disinhibited social engagement disorder symptoms

**DOI:** 10.1016/j.ynirp.2026.100323

**Published:** 2026-01-23

**Authors:** Shoko Shimada, Toshiki Iwabuchi, Motofumi Sumiya, Koji Shimada, Shinichiro Takiguchi, Kai Makita, Akiko Yao, Takashi X. Fujisawa, Atsushi Senju, Akemi Tomoda

**Affiliations:** a United Graduate School of Child Development, The University of Osaka, Kanazawa University, Hamamatsu University School of Medicine, Chiba University, and University of Fukui, Yamadaoka, Suita, Osaka, 565-0871, Japan; b Department of Psychiatry, Kagoshima University Graduate School of Medical and Dental Sciences, 8-35-1, Sakuragaoka, Kagoshima-shi, Kagoshima, 890-0075, Japan; c Research Center for Child Mental Development, Hamamatsu University School of Medicine, 1-20-1 Handayama, Chuo-ku, Hamamatsu, Shizuoka, 431-3192, Japan; d Department of Clinical Psychology, Hanazono University, 8-1, Nishinokyo, Tsubonouchi-cho, Nakagyo-ku, Kyoto, 604-8456, Japan; e Research Center for Child Mental Development, University of Fukui, 23-3 Matsuokashimoaitsuki, Eiheiji-cho, Yoshida-gun, Fukui, 910-1193, Japan; f Department of Child and Adolescent Psychological Medicine, University of Fukui Hospital, 23-3 Matsuokashimoaitsuki, Eiheiji-cho, Yoshida-gun, Fukui, 910-1193, Japan; g Graduate School of Intercultural Studies, Kobe University, 2-1, Rokkoudai-cho, Nada-ku, Kobe, Hyogo, 657-8501, Japan

**Keywords:** Residential care, fMRI, Functional connectivity, Neurodevelopmental resilience, RAD, DSED

## Abstract

Adverse childhood experiences are a risk factor for attachment disorders. While several neuroimaging studies have shown changes in functional networks in children who have experienced institutional care, the results are inconsistent. Furthermore, no research has been conducted on how structured residential care, such as Japan's small-group family-style care, influences attachment-related symptoms and functional connectivity. This study compared attachment-related symptoms (reactive attachment disorder [RAD] and disinhibited social engagement disorder [DSED] symptoms) between youth aged 9–18 years raised in Japanese small-group residential care (RC; n = 31) and those raised in birth families but not in RC (NRC; n = 37). Group differences in resting-state functional connectivity were also analyzed using multivariate pattern analysis (MVPA) on functional magnetic resonance imaging (fMRI) data. MVPA revealed group differences in whole-brain functional connectivity patterns from the right occipital pole and the left lingual gyrus (LLG). Functional connectivity between the LLG and the frontal medial cortex (FMC) was reduced in RC youth. LLG-FMC connectivity was positively correlated with RAD scores, while longer duration of stay in RC was negatively correlated with RAD symptoms. This study highlights caregiving environment's influence on attachment-related symptoms and functional connectivity, higher levels of RAD and DSED symptoms and reduced LLG-FMC functional connectivity in the RC group. However, this study further demonstrated not only the association between longer stays in family-like RC and the reduction of RAD symptoms but also changes in the connectivity. These findings suggest that stable, high-quality care may have the potential to mitigate adverse developmental outcomes.

## Highlights

• Youth in residential care (RC) showed higher RAD and DSED symptoms.
• Whole-brain functional connectivity (FC) patterns were altered in RC youth.
• Reduced FC in RC youth was positively correlated with RAD symptoms.
• Longer RC durations were negatively correlated with RAD symptoms.

## 1 Introduction

Millions of children worldwide are institutionalized, a circumstance that significantly impacts their developmental trajectories, often resulting in attachment disorders (van Ijzendoorn et al., 2020; Zeanah and Gleason, 2015). Evidence linking institutionalization to attachment disorders largely stems from two major longitudinal studies focused on severely deprived institutional environments: the English and Romanian Adoptees (ERA) Study (O'Connor et al., 1999; Sonuga-Barke et al., 2017) and the Bucharest Early Intervention Project (BEIP) (Smyke et al., 2002; Zeanah et al., 2017). Findings from these studies underpin the definitions in the fifth edition of the Diagnostic and Statistical Manual of Mental Disorders (DSM-5) (American Psychiatric Association, 2013) for two attachment disorders that share a history of extremely insufficient caregiving during childhood as a diagnostic criterion: reactive attachment disorder (RAD) and disinhibited social engagement disorder (DSED). RAD is characterized as an internalizing disorder with depressive symptoms and withdrawal, while DSED involves disinhibition and externalizing behaviors. Symptoms of RAD and DSED can persist into adolescence and early adulthood, leading to a significant personal and societal burden. Notably, DSM-IV classified the features of RAD and DSED as subtypes of a single disorder, whereas DSM-5 conceptualizes RAD and DSED as separate disorders, each characterized by unique diagnostic criteria and clinical presentations, reflecting advances in the understanding of their developmental trajectories and underlying etiologies. The ERA and BEIP studies revealed elevated RAD and DSED symptoms in youth living in institutions. However, the neural correlates of these attachment disorders remain underexplored in this population.

In terms of neurobiological mechanisms, atypical development of functional networks in the brain is believed to underlie developmental challenges in institutionalized children, including increased RAD and DSED symptoms. Studies using electroencephalography (EEG) from the BEIP have demonstrated altered functional networks in institutionalized children (Debnath et al., 2020; Wade et al., 2019). Compared to never-institutionalized children, those who experienced institutionalization showed lower alpha power during rest (Debnath et al., 2020), and higher alpha power at 8 years of age was associated with better executive function in later developmental stages (Wade et al., 2019). Additionally, several studies using resting-state functional magnetic resonance imaging (rsfMRI) have examined differences in functional connectivity between previously institutionalized and never-institutionalized youth. This imaging method assesses the strength of functional connections between brain regions with high spatial resolution (Fareri et al., 2017; Herzberg et al., 2021). Fareri et al. (2017) reported that the ventral striatal-medial prefrontal cortex (mPFC) functional connectivity mediated the relationship between institutionalization and social problems in youth. Similarly, Herzberg et al. (2021) found increased functional connectivity between the amygdala and ventral mPFC in previously institutionalized young adults compared to never institutionalized peers. Although these findings suggest altered functional networks in children exposed to institutionalization, the results are mixed. One contributing factor may be the reliance on a priori-defined regions of interest in previous studies. Thus, exploring the neural mechanisms underlying RAD and DSED symptoms in institutionalized youth with unbiased analytical methods free from a priori assumptions is crucial.

In addition, the quality of institutional care is a critical factor in understanding the mechanisms underlying RAD and DSED symptoms in institutionalized youth (Giraldi et al., 2022). While the previously mentioned studies have largely focused on extreme situations, such as the ERA study and BEIP, these severely deprived conditions are not representative of residential care (RC) in developed countries like Japan. Gunnar (2001) categorized institutions into three levels based on the quality of caregiving: (1) facilities characterized by global deprivation of a child's health, nutrition, stimulation, and relational needs; (2) facilities providing adequate health and nutritional support but depriving children of stimulation and relationship needs; and (3) facilities that meet all needs except for a stable, long-term relationship with a consistent caregiver. Building on this framework, van Ijzendoorn et al. (2011) proposed a fourth level of caregiving, characterized by a stable and consistent caregiving environment of the highest quality. In Japan, governmental reforms have promoted a transition from traditional institutional forms to small-group, family-like RC homes. According to the definition by Ministry of HealthLabour and Welfare (2017), this type of RC home is characterized by several key requirements: (1) a small living unit accommodating a maximum of six children, (2) consistent caregiving by multiple caregivers who are always present when the children are in the facility, and (3) minimal changes in caregiving staff. Japanese small-group, family-like RC homes are designed to provide stable and consistent relationships with caregivers, aligning with the fourth level of institutional caregiving proposed by van Ijzendoorn et al. (2011). These RC homes also offer therapeutic care and treatment with the support of trained caregivers (Cantwell et al., 2012; Porter et al., 2020; Steels and Simpson, 2017), which can benefit youth exposed to childhood adversities. Consequently, the duration of time spent in these RC homes may be associated with alleviated attachment problems. Unlike institutions in extreme situations studied in the ERA and BEIP research, Japanese small-group family-like RC homes, which integrate both living and therapeutic functions, may reduce RAD and DSED symptoms while also altering neural correlates in youth.

In the current study, we investigated Japanese youth living in small-group, family-like RC to address three major gaps in understanding the relationship between institutionalization and attachment problems. First, using the RAD and DSED Assessment (RADA) (Lehmann et al., 2018), which effectively measures RAD and DSED symptoms based on DSM-5 criteria, we examined whether Japanese youth living in RC exhibited higher RAD and DSED symptoms than those who had never lived in residential care (NRC). Second, to explore the neural mechanisms underlying attachment problems in youth in RC, we investigated differences in resting-state functional connectivity (rsFC) between the RC and NRC groups. Given the limited number of previous studies and their mixed results, potentially due to varying definitions of regions of interest, multivariate pattern analysis (MVPA) was adopted. This assumption-free analytical method detects intrinsic functional connectivity patterns (Nieto-Castanon, 2022) and unbiasedly identifies candidate seed regions showing differential whole-brain connectivity between groups. Third, to examine the effect of the duration spent in RC within the Japanese system and differential functional connectivity patterns on RAD/DSED symptoms, we conducted multiple regression analysis in the RC group.

## 2 Materials and methods

### 2.1 Participants

Thirty-one youth aged 9–18 years living in family-like RC (14 females; mean age ± standard deviation: 13.14 ± 2.87 years) and 37 youth from NRC (16 females; mean age: 13.13 ± 3.01 years) participated in this study. Regarding sex information for each participant, a binary sex categorization assigned at birth was reported by their caregivers or parents. Participants in both groups were excluded if they had an intellectual disability (full-scale intelligence quotient [FSIQ] < 70), a history of brain injury or neurological disorders (e.g., epilepsy), MRI contraindications, or possible pregnancy. The RC group included two participants with neurodevelopmental disorders (1 with attention-deficit/hyperactivity disorder [ADHD], 1 with autism spectrum disorder [ASD] + ADHD), while the NRC group included one participant with ADHD. Regarding handedness, the RC group included five left-handed and one ambidextrous participant, while the NRC group included one left-handed participant. After data acquisition, participants whose fMRI data contained more than 20 % outliers were excluded from further analyses (details provided in the Neuroimaging Preprocessing section). The final sample consisted of 28 RC youth (14 females; mean age: 13.04 ± 2.73 years) and 33 NRC youth (15 females; mean age: 13.12 ± 2.90 years) (Table 1). The two groups were matched for sex (*t*(57.262) = 0.349, *p* = 0.729) and age (*t*(58.29) = −0.116, *p* = 0.908). However, a significant difference was observed in FSIQ, with RC youth showing lower FSIQ than NRC youth (*t*(57.42) = −4.957, *p* < 0.001). FSIQ was estimated using the Wechsler Intelligence Scale for Children, Fourth Edition (WISC-IV) (Wechsler, 2003) or the Wechsler Adult Intelligence Scale Third Edition (WAIS-III) (Wechsler, 1997).

**Table 1:** Group comparisons of demographic and behavioral information.

	Non-residential care (N = 33)	Residential care (N = 28)	Statistics	P-value
Female (%)	45.55	50.00	0.349	0.729
Age (years, mean ± SD)	13.12 ± 2.90	13.04 ± 2.73	−0.116	0.908
FSIQ (mean ± SD)	106.03 ± 11.27	93.54 ± 8.02	−4.957	<0.001
Residential care duration (years, mean ± SD)	–	5.59 ± 3.90	–	–
Age of separation from birth family	–	7.98 ± 4.78	–	–
Abuse or Neglect (%)	–	85.71	–	–
Abuse (%)	–	64.29	–	–
Neglect (%)	–	67.86	–	–
Diagnosis of ASD	0	1	1.198	0.273
Diagnosis of ADHD	1	1	0.547	0.459
Psychotropic Medication (N)	–	Methylphenidate (2)	–	–
Handedness	1 left-handed	5 left-handed and 1 ambidextrous	5.143	0.076
RADA: RAD symptoms (mean ± SD)	0.39 ± 0.69	1.96 ± 3.03	2.636	0.013
RADA: DSED symptoms (mean ± SD)	0.40 ± 1.25	2.04 ± 3.01	2.650	0.012

*Abbreviations*: FSIQ, full-scale intelligence quotient; SD, standard deviation; RAD, reactive attachment disorder; DSED, disinhibited social engagement disorder; ASD, autism spectrum disorder; ADHD, attention deficit hyperactivity disorder; RADA, reactive attachment disorder assessment.

*Note*: The Statistics column reports *t*-values for comparisons of continuous variables and chi-square values for comparisons of categorical variables.

For RC youth, the average time spent in residential care was 5.59 years (SD = 3.90 years), and their average age of separation from their birth family was 7.98 years (SD = 4.78 years). Based on official records provided by the local child guidance center, 85.71 % of the youth had experienced maltreatment (abuse and/or neglect) before being placed in RC. All RC youth had ongoing contact with their families of origin as part of the family reintegration process. One author (SS) checked the medication status of each participant, and two youths underwent a psychotropic medication washout period of at least two days before the examination. This washout period has been commonly used in neuroimaging literature (Harkness et al., 2024).

In 2020, we contacted RC homes in Fukui prefecture, Japan, to invite their participation in this study. Of the five RC homes contacted, two directors responded positively, provided written informed consent, and permitted their youth to participate in this study. Written informed consent or assent was also obtained from each youth and their legal guardian. NRC youth were recruited from the local community. The study protocol was approved by the Research Ethics Committee of the University of Fukui (20210004) and conducted in accordance with the Declaration of Helsinki and the Ethical Guidelines for Medical and Health Research Involving Human Subjects of Japan.

### 2.2 Behavioral measures

All participants completed the RAD and DSED Assessment (RADA) (Lehmann et al., 2018), a standardized assessment tool aligned with the DSM-5 criteria for RAD (11 items) and DSED (9 items) symptoms. For this study, the RADA was translated into Japanese and back-translated into English by multiple editors at an academic translation company (Ulatus, Crimson Interactive Pvt. Ltd.; https://www.ulatus.jp/). We obtained translation permission from the original authors. One of the authors (SS) reviewed the original and back-translated versions to confirm equivalence and made minor modifications to the wording to optimize the tool for interviewing. SS also conducted the RADA through face-to-face, semi-structured interviews with caregivers in RC and parents in NRC. Responses to each item were coded on a 3-point scale (0 = No, 1 = A little, 2 = A lot), resulting in scale scores ranging from 0 to 22 for RAD and 0 to 18 for DSED (Lehmann et al., 2018). We calculated Cronbach's alpha for the RAD and DSED scores to assess the internal consistency of the translated scale and employed a two-sample *t*-test to compare the RAD and DSED scores between the two groups. The Japanese version of the RADA is available for research purposes upon reasonable request to the corresponding author and with permission from the original developers.

### 2.3 MRI data acquisition

All participants were scanned using a 3T MRI scanner (Signa PET/MR; GE Healthcare, Milwaukee, WI, USA) equipped with an eight-channel head coil. Functional imaging was performed using a T2∗-weighted gradient-echo echo-planar imaging sequence, producing 40 continuous transaxial slices with a thickness of 3.5 mm and a 0.5 mm gap, covering the entire cerebrum and cerebellum (repetition time [TR] = 2300 ms; echo time [TE] = 30 ms; flip angle [FA] = 81°; field of view [FOV] = 192 mm; 64 × 64 matrix; voxel dimensions = 3.0 × 3.0 × 4.0 mm). During resting-state image acquisition, participants were instructed to lie still and relax while viewing a fixation cross. A total of 196 vol were acquired per participant. Additionally, high-resolution structural images were obtained using a 3D T1-weighted fast spoiled-gradient recalled imaging sequence (TR = 8.48 ms; TE = 3.24 ms; FA = 11°; FOV = 256 mm; 256 × 256 matrix; 172 slices; voxel dimensions = 1.0 × 1.0 × 1.0 mm).

### 2.4 Neuroimaging preprocessing

Preprocessing and statistical analyses of resting-state MRI data were conducted using the CONN toolbox (version 20b) (Whitfield-Gabrieli and Nieto-Castanon, 2012) and SPM 12 (7771) on MATLAB 2021a (MathWorks, Natick, MA, USA). To stabilize magnetization, the first six volumes were discarded, leaving 190 volumes for analysis. Preprocessing of functional scans included spatial realignment and unwarping, slice-time correction, and outlier detection using ART-based scrubbing with thresholds of 0.9 mm framewise displacement (FD) and five standard deviations in global BOLD signal changes. Seven participants (4 NRC, 3 RC) were excluded from the analysis due to more than 20 % of their data points being flagged as outliers (mean FD, 0.19 mm for the RC group and 0.13 mm for the NRC group; mean percentage of scrubbed volumes, 3.31 % for the RC group and 1.07 % for the NRC group). For normalization, Diffeomorphic Anatomical Registration Through Exponentiated Lie algebra (DARTEL) was used to create a study-specific anatomical template for the youth participants (Ashburner, 2007). Segmented gray and white matter images obtained through a unified segmentation approach were transformed into a common coordinate space using the DARTEL registration algorithm to create the template. This study-specific template was then affine-normalized to the Montreal Neurological Institute (MNI) space with the ICBM Probabilistic Atlases. The DARTEL registration and normalization parameters were applied to each functional image and T1-weighted anatomical image. Normalized functional images were smoothed with a Gaussian kernel of 8 mm full width at half maximum (FWHM).

In addition to the preprocessing pipeline, the CONN toolbox implemented a rigorous denoising process, including removal of physiological noise components using the aCompCor method, scrubbing, and temporal band-pass filtering. During these processes, we used the default setting in the CONN toolbox. Global signal regression was not applied. Physiological noise signals from white matter (5 components) and cerebrospinal fluid (5 components) were regressed out. Outliers detected by the scrubbing procedure (≤37 components) and 12 potential noise components (six rigid-body parameters and their temporal derivatives) were also regressed to minimize motion-related artifacts. Temporal band-pass filtering was applied at 0.008–0.09 Hz.

### 2.5 Multivariate pattern analysis

Multivariate pattern analysis (MVPA) was performed using the CONN Toolbox (Nieto-Castanon, 2022; Whitfield-Gabrieli and Nieto-Castanon, 2012) to unbiasedly identify candidate seed regions showing differential whole-brain functional connectivity patterns between groups (RC and NRC). At the first individual level, features of interest were extracted using 64 components derived from principal component analysis. At the group level, an omnibus *F*-test was performed using 10 MVPA components to evaluate the RC > NRC contrast. The first 10 components were selected to maintain a participant-to-component ratio of approximately 5:1 (Whitfield-Gabrieli and Nieto-Castanon, 2012). In the group-level model, FSIQ and mean head motion during the scan were included as regressors of no interest. Clusters that passed both a voxel-level threshold of *p* < 0.001 and a false discovery rate (FDR)-corrected cluster-level threshold of *p* < 0.05 were selected for post-hoc analysis, as detailed in the following section.

### 2.6 Post-hoc characterization of MVPA-derived cluster

To investigate between-group differences in rsFC, a post-hoc whole-brain seed-to-voxel analysis was performed using the MVPA-derived clusters of interest. Pearson's correlation coefficients were calculated between the MVPA seed-time course and the time courses of all other voxels in the brain. These coefficients were then transformed into normally distributed z-scores using Fisher's *r*-to-*z* transformation for second-level general linear model analyses. A two-tailed *t*-test was used to compare the RC and NRC groups, employing a voxel-level threshold of p < 0.001 for the whole brain and an FDR-corrected cluster threshold of *p* < 0.05. As the seed regions were defined using the MVPA on the same dataset, this post-hoc analysis must be considered exploratory and descriptive. To assess the robustness of the between-group difference, we performed a jackknife analysis and a bootstrap analysis (see Supplementary Methods).

### 2.7 Multiple linear regression analysis on RAD and DSED scores

Multiple linear regression analysis was conducted to examine whether rsFC differences between groups and the duration of time spent in RC were associated with RAD and DSED symptoms within the RC group. This analysis focused on the RC group to avoid potential circularity. Since significant group differences were observed in both the rsFC values and the RAD/DSED scores, an analysis using data from all the participants could have led to an overestimation of the associations between these values. Independent variables included age, the duration spent in residential care, and rsFC values after Fisher's r-to-z transformation from regions showing significant group differences in the post-hoc seed-to-voxel analysis. The model was fitted using the lm_robust command from the “estimatr” package (Blair et al., 2018), which employs heteroskedasticity-consistent (HC0) robust standard errors. To control the family-wise error for the relevant hypothesis tests, a Bonferroni adjustment across the two models was employed (0.025). However, to avoid being overly conservative, multiple testing corrections for the coefficients in each model were not applied. To ensure model robustness, multicollinearity among regressors was assessed using the “performance” package (Lüdecke et al., 2021). The Shapiro–Wilk normality test was conducted to evaluate whether residuals adhered to the assumption of normally distributed errors (Williams et al., 2013), and the normality of residuals was confirmed. Additionally, we performed standard influential diagnostics to evaluate the impact of a few extreme observations and conducted a sensitivity analysis that excluded these observations (see Supplementary Methods). All analyses were performed using R software (version 4.2.0; R Core Team, 2021), an open-source environment for statistical computing.

### 2.8 Sensitivity analyses for rsfMRI data

To confirm the robustness of the MVPA findings, we performed a sensitivity analysis employing a different smoothing kernel (6 mm), a stricter scrubbing threshold (FD ≥ 0.5 mm), and regressing out Friston 24 head-motion parameters, along with aCompCor and outliers in the denoising processing (Friston et al., 1996) (Sensitivity Analysis 1). Additionally, to check the possibility that the main MVPA result was affected by the inclusion of the FSIQ as a covariate, we performed the MVPA without the FSIQ (Sensitivity Analysis 2). In these sensitivity analyses, we used the same statistical threshold (a voxel-level threshold of *p* < 0.001 and an FDR-corrected cluster-level threshold of *p* < 0.05). If the results were not replicated at this threshold, we progressively applied more lenient thresholds (e.g., a voxel-level threshold of *p* < 0.005 and an FDR-corrected cluster-level threshold of *p* < 0.05).

## 3 Results

### 3.1 Group comparisons of behavioral characteristics

Internal consistency was acceptable for both RAD (α = 0.716) and DSED (α = 0.768). A comparison of RADA scores revealed that the RC group exhibited higher levels of both RAD and DSED symptoms than the NRC group (RAD symptoms, *t*(29.393) = 2.636, *p* = 0.013; DSED symptoms, *t*(34.876) = 2.650, *p* = 0.012), indicating greater psychological difficulties in these domains.

### 3.2 MVPA results

**Fig. 1:**
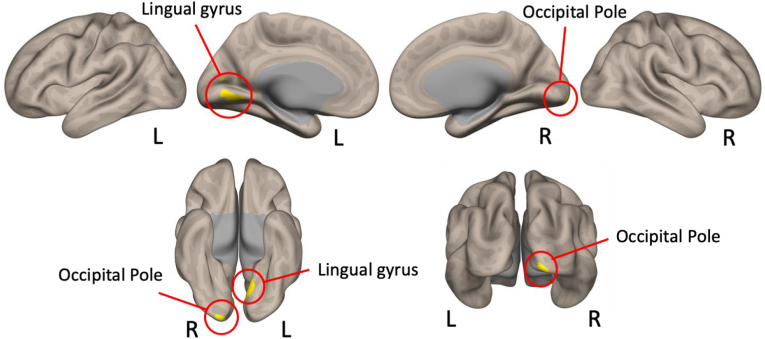
Whole-brain MVPA connectivity differences. The right occipital pole (peak coordinate, [16, −100, −16]; cluster size, 76) and the left lingual gyrus (peak coordinate, [−2, −70, 0]; cluster size, 64) showed significant group differences in connectivity patterns with the rest of the brain when comparing the two groups. MVPA, multivariate pattern analysis; L, left; R, right.

Statistically significant clusters identified through whole-brain connectome-wide MVPA are presented in Fig. 1 and Table 2 (voxel-level *p* < 0.001 with an FDR-corrected cluster-level threshold of *p* < 0.05). Brain regions were anatomically labeled using the built-in atlas in the CONN toolbox. The first 10 MVPA components accounted for 93.72 % and 87.38 % of the variance in gray and white matter, respectively. The results indicate that whole-brain functional connectivity patterns from the right occipital pole (ROP) (peak cluster MNI coordinates: 16, −100, −16) and the left lingual gyrus (LLG) (peak cluster MNI coordinates: −2, −70, 0) were significantly different between groups.

**Table 2:** Whole-brain MVPA results indicating the regions that show significant group differences in connectivity patterns with the rest of the brain.

	Brain Region	Peak cluster coordinates (MNI)	Voxels per cluster
Cluster 1:	Occipital pole right	16 –100 −16	76
Cluster 2:	Lingual gyrus left	−2 −70 0	64

*Abbreviations:* MNI, Montreal Neurological Institute; MVPA, multivariate pattern analysis.

### 3.3 Post-hoc seed-to-voxel analysis of MVPA-derived clusters of interest

**Fig. 2:**
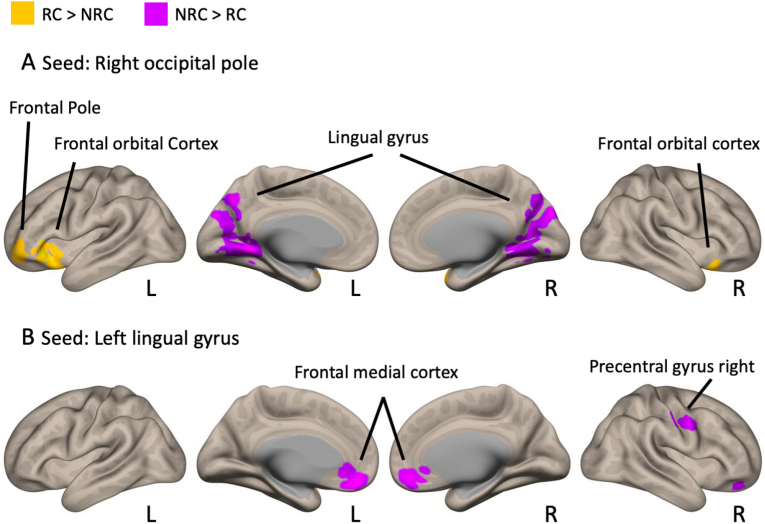
Seed-to-voxel rsFC analysis for RC vs NRC. Results from second-level seed-to-voxel rsFc analysis for RC vs NRC contrast for MVPA-derived cluster. Yellow regions show higher connectivity for the RC group compared to the NRC group. Violet regions show the opposite pattern. For the right occipital pole seed, the left lingual gyrus (peak coordinate, [−14, −54, −4]; cluster size, 4163), left frontal orbital cortex (peak coordinate, [−42, 26, −4]; cluster size, 622), left frontal pole (peak coordinate, [−38, 54, −10]; cluster size, 417), and right frontal orbital cortex (peak coordinate, [36, 20, −20]; cluster size, 204) were identified. For the left lingual gyrus seed, the frontal medial cortex (peak coordinate, [−6, 44, −18]; cluster size, 736) and right precentral gyrus (peak coordinate, [62, −4, 42]; cluster size, 294) were identified. MVPA, multivariate pattern analysis; rsFC, resting-state functional connectivity; RC, residential care; NRC, non-residential care; L, left; R, right.

The results of the post-hoc seed-to-voxel analysis using the MVPA-derived clusters (ROP and LLG) are presented in Table 3 and Fig. 2. For the ROP seed, the RC group exhibited weaker functional connectivity with the bilateral lingual gyrus (BLG) and stronger functional connectivity with the left frontal pole (LFP) and the left and right frontal orbital cortices (LFOC and RFOC) than the NRC group (Fig. 2A). For the LLG seed, the RC group showed weaker functional connectivity with the frontal medial cortex (FMC) and the right precentral gyrus (RPG) than the NRC group (Fig. 2B). Both jackknife and bootstrap analyses supported the robustness and stability of the group differences in these rsFC values (see Supplementary Results).

**Table 3:** Results from second-level seed-to-voxel rsFC analysis for RC > NRC contrast for MVPA-derived cluster.

Brain region	Peak cluster coordinates (MNI)	Voxels per cluster	T-value
Seed: Right occipital pole			
Lingual gyrus left	−14 −54 −4	4163	−6.44
Frontal orbital cortex left	−42 26 −4	622	6.24
Frontal pole left	−38 54 −10	417	5.66
Frontal orbital cortex right	36 20 −20	204	5.08

Seed: Left lingual gyrus			
Frontal medial cortex	−6 44 −18	736	−4.54
Precentral gyrus right	62 −4 42	294	−4.38

*Abbreviations*: rsFC, resting-state functional connectivity; RC, residential care; NRC, non-residential care; MNI, Montreal Neurological Institute; MVPA, multivariate pattern analysis.

*Note*: Positive *t*-values indicate higher connectivity for the RC group compared with the NRC group, while negative *t*-values indicate the opposite pattern.

### 3.4 Multiple linear regression analysis on RAD and DSED scores

**Fig. 3:**
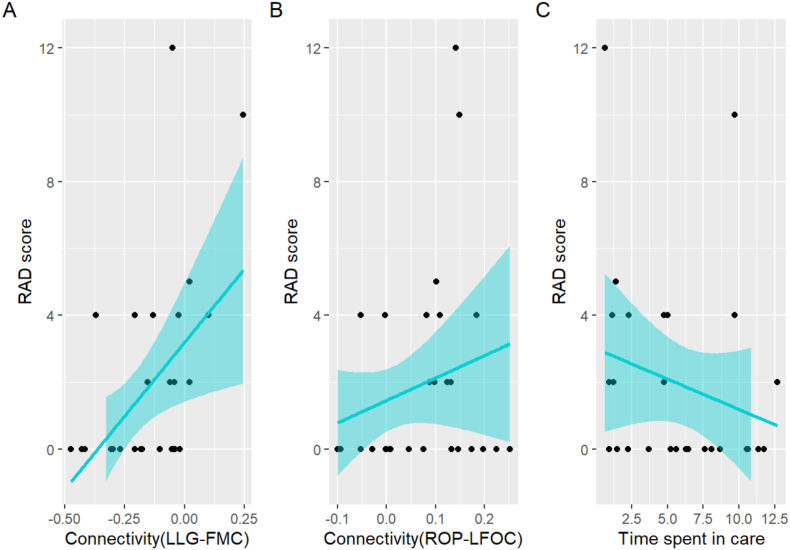
Functional connectivity and time in care impact on RAD scores. Influence of functional connectivity (A and B) and time spent in care (C) on RAD score. RAD, reactive attachment disorder; LLG, left lingual gyrus; FMC, frontal medial cortex; ROP, right occipital pole; LFOC, left frontal orbital cortex.

We investigated whether the functional connectivity patterns derived from the seed-to-voxel analysis were associated with attachment symptoms (RAD and DSED scores) using regression analyses within the RC group. The regression model was statistically significant for RAD (adjusted *R*^2^ = 0.384, *F*(8, 19) = 4.863, *p* = 0.002, Bonferroni-corrected *p* = 0.004) but not for DESD (adjusted *R*^2^ = −0.168, *F*(8, 19) = 0.952, *p* = 0.500). For the RAD score (Fig. 3 and Supplementary Table 1), a significant positive association was observed for LLG-FMC connectivity (*β* = 6.810, *p* = 0.003, Bonferroni-corrected *p* = 0.006). We also found a significant positive association with the RAD score and ROP-LFOC connectivity, but this did not survive a multiple comparison correction (*β* = 10.583, *p* = 0.029, Bonferroni-corrected *p* = 0.058). Other connectivity patterns, including LLG-RPG (*β* = 2.011, *p* = 0.452), ROP-BLG (*β* = −12.014, *p* = 0.060), ROP-LFP (*β* = −3.893, *p* = 0.298), and ROP-RFOC (*β* = −5.648, *p* = 0.296), were not significant. Additionally, the time spent in RC was significantly negatively associated with RAD scores (*β* = −0.286, *p* = 0.024, Bonferroni-corrected *p* = 0.048), while age showed no significant effect (*β* = 0.166, *p* = 0.425). For the DSED score, none of the regressors showed a significant relationship (Supplementary Table 1). Influence diagnostics and sensitivity analyses confirmed the robustness of these results, which were not driven by a few data points (see Supplementary Results).

### 3.5 Results of sensitivity analyses for rsfMRI data

Sensitivity Analyses 1 and 2 showed that the nearly identical regions were identified by the group comparison only when a more liberal threshold was applied (a voxel-level threshold of *p* < 0.005 and an FDR-corrected cluster-level threshold of *p* < 0.05; see Supplementary Results, Supplementary Figs. 1 and 2, and Supplementary Tables 3 and 4). These results generally supported the original findings, but given the need for a more liberal threshold, caution is required when interpreting the robustness of the results.

## 4 Discussion

This study identified three novel findings. First, using the RADA assessment tool aligned with DSM-5 criteria, we demonstrated that youth living in Japanese family-like RC homes exhibited higher RAD and DSED symptom scores than youth who had never been in RC. These findings suggest that, despite the relatively supportive caregiving environment of family-like RC homes, institutionalized youth continue to experience heightened attachment-related symptoms. These elevated scores likely reflect the enduring psychological impact of pre-placement adversities, such as maltreatment or neglect. Second, we employed MVPA to unbiasedly identify between-group differences in whole-brain functional connectivity patterns. This analysis revealed significant group differences in whole-brain functional connectivity patterns originating from the ROP and the LLG. Third, within the RC group, multiple linear regression analyses revealed complex relationships between functional connectivity patterns, caregiving duration, and RAD/DSED symptoms. While the RC group exhibited weaker functional connectivity between the LLG and the FMC than the NRC group, we observed a significant positive correlation between LLG-FMC connectivity and RAD scores within the RC group. Conversely, longer durations in family-like RC homes were negatively correlated with RAD scores, suggesting a potential therapeutic effect of extended caregiving in reducing attachment-related symptoms.

To the best of our knowledge, this is the first study to compare RAD and DSED symptoms using the RADA, an assessment tool aligned with DSM-5 criteria, between youth living in RC homes (RC group) and youth living with their birth families who have never been in RC (NRC group). By employing the RADA to quantify and assess symptoms rather than to diagnose RAD and DSED, we observed higher scores in RAD and DSED symptoms among youth living in RC homes than those in the NRC group. Notably, most youth in the RC group entered these facilities following disruptions in familial care rather than immediately after birth. These findings suggest that adversities experienced prior to RC placement, such as neglect and unstable parent-child relationships within their birth families, likely contributed to the elevated RAD and DSED symptoms observed in the RC group. This highlights the potential long-term impact of early-life adversities on attachment-related behaviors, both in the RC group studied here and potentially in youth residing in RC more broadly.

From a neurophysiological perspective, rsfMRI analysis revealed distinct whole-brain functional connectivity patterns originating from the ROP and LLG between the RC and NRC groups. These regions, part of the visual cortex, have been linked to the effects of adversity in prior structural MRI studies. For instance, youth who witnessed domestic violence during childhood demonstrated reduced cortical thickness in the occipital pole and lingual gyrus, as well as decreased gray matter volume in the lingual gyrus (Tomoda et al., 2012). Reduced cortical thickness in the lingual gyrus was also associated with the severity of PTSD symptoms in a structural MRI study (Wrocklage et al., 2017). Due to variations in the types of adverse experiences or target clinical conditions and differences in imaging modalities (i.e., structural and functional MRI), comparisons between the present study and prior studies must be made with caution. Nevertheless, our findings do not contradict the idea that adverse experiences could affect some regions related to visual processing. Additionally, the hemispheric asymmetry of our results may reflect different mechanistic pathways through which childhood adversity affects the left and right sides of the brain. It has been suggested that positive and negative emotional stimuli are processed by different cerebral hemispheres, and the effects of adverse experiences on brain structure and function may manifest differently in the left and right hemispheres, as discussed by Shimada et al. (2015) and Nakagawa et al. (2021). While we cannot draw definitive conclusions about lateralization based on this result, it could be an interesting avenue for future research.

Changes in sensory cortical regions, such as the visual cortex, are theorized to represent specific adaptations aimed at mitigating distress, as proposed by Teicher et al. (2016). Supporting this theory, task-based functional imaging studies have consistently reported alterations in brain activity within the occipital pole and lingual gyrus during various tasks in children exposed to adversity (Bennett et al., 2009; Birn et al., 2017; Bruce et al., 2013; Gonzalez et al., 2016; Holz et al., 2017; Just et al., 2019; Lee et al., 2015; Malisza et al., 2012; Miller et al., 2015; Müller et al., 2013; Norman et al., 2013; Sheinkopf et al., 2009; Suzuki et al., 2014). Consistent with these studies, our rsfMRI findings newly demonstrated that the ROP and LLG, components of the visual cortex, exhibited distinct whole-brain functional connectivity patterns between the RC and NRC groups. These findings support the hypothesis that adverse experiences lead to neural adaptations in the visual cortex, which may influence everyday information processing, as theorized by Teicher et al. (2016).

Using the ROP and LLG clusters obtained from MVPA as seed regions, a post-hoc analysis further illustrated the rsFC differences between the groups. In addition to reduced functional connectivity within the occipital cortex (i.e., ROP and BLG) in the RC group, both occipital seed regions exhibited altered connectivity patterns with the frontal cortex in the RC group. Specifically, the ROP showed connectivity changes with the LFP and bilateral frontal orbital cortex, while the LLG demonstrated altered connectivity with the FMC and the RPG. These results are consistent with previous studies reporting reduced white matter integrity in the inferior fronto-occipital fasciculus, a key tract connecting the frontal and occipital lobes in children who have experienced adversity (Frodl et al., 2012; Hanson et al., 2013; Huang et al., 2012; Lim et al., 2019; Tendolkar et al., 2018; Ugwu et al., 2015). Reduced integrity in this pathway may underlie the disrupted functional connectivity observed between the frontal and occipital cortices in the RC group. Future research should explore the role of functional connectivity within and between the visual cortices, with a particular focus on the frontal-occipital connections, to further elucidate the neural consequences of childhood adverse experiences. These investigations could provide insights into the mechanisms driving altered cognitive and emotional processing in this population. However, given the exploratory nature of the post-hoc analysis, these results must be interpreted carefully. This analysis was based on the seed clusters obtained from the MVPA analysis using the same dataset. Although we confirmed the robustness of the group differences in these rsFC values, future studies must replicate these results using an independent dataset.

Within the RC group, higher RAD scores were associated with greater functional connectivity between the LLG and FMC. Intuitively, given that the RC group exhibited reduced LLG-FMC connectivity overall, one might expect weaker LLG-FMC connectivity to correlate with higher RAD symptoms. In other words, a negative correlation between LLG-FMC connectivity and RAD symptoms would seem more likely; however, the results showed the opposite pattern. This unexpected finding is particularly intriguing. One possible explanation is that the reduced functional connectivity between the LLG and FMC in the RC group reflects an adaptive or compensatory change in the brain network of individuals who have experienced childhood adversity. If weaker LLG-FMC connectivity serves an adaptive role, it could plausibly contribute to lower RAD symptoms in youth who entered RC facilities after experiencing adversity. Both individual and environmental factors, such as innate resilience or the influence of high-quality caregiving in small-group family-like RC settings, may facilitate these adaptive changes. Notably, RAD scores were negatively correlated with the time spent in care, suggesting that longer stays in RC facilities were associated with lower RAD symptoms. This finding aligns with the adolescent recalibration hypothesis (Colich et al., 2021; Gunnar et al., 2019), which posits that during adolescence, the effects of past adversity may be recalibrated or mitigated. Given that this study targeted youth aged 9–18 years, it is plausible that living in RC facilitated recovery from adverse experiences. High-quality care in small-group family-like RC environments may have promoted adaptive changes in brain networks, reducing the impact of early adversity as a risk factor for attachment disorders.

It is important to acknowledge the limitations of this study. First, although acceptable internal consistency was confirmed for the Japanese version of RADA (RAD: α = 0.716; DSED: α = 0.768), it should be noted that it has yet to undergo formal psychometric validation. Furthermore, the finalization of the translation and the actual interviews were conducted by the same researcher (SS), which may have introduced an interviewer bias. Inter-rater reliability should be assessed in future studies. Second, the RADA interviews were conducted with the primary caregiver of each child, which may have introduced bias. The caregiver's personality, level of involvement, and subjective interpretations of the child's behavior may have influenced the scores to some extent. This differs from observational methods and may have affected the accuracy of the assessment. The discrepancy in the characteristics of the respondents (i.e., caregivers for the RC group vs. parents for the NRC group) may also have resulted in systematic bias. Third, the small sample size limits the generalizability of the findings of this study regarding, for example, biological sex and types of maltreatment. Fourth, while it would have been ideal to include all youth residing in the participating facilities at the time of sampling, participation required consent from their legal guardians. Consequently, the sample may have been biased toward youth who maintained relatively more interaction with their legal guardians even after entering RC facilities, potentially limiting the representativeness of the findings for the broader RC population. Fifth, although multiple robustness and sensitivity analyses (robust regression, quantile regression, and influence diagnostics) generally supported the consistency of the main associations, the within-group regression results should still be interpreted with caution due to the modest sample size and potential instability of secondary covariates. The key effects (e.g., LLG-FMC and duration) remained directionally and materially stable, whereas several non-significant ROP-related predictors showed higher sensitivity to influential observations. The non-uniform distribution of FC values and the limited number of high-leverage observations may have reduced the precision of coefficient estimates. Future studies using larger and more balanced samples are needed to confirm the robustness and generalizability of these effects. Lastly, this study's cross-sectional design limits its ability to infer causality or track changes over time. While the findings provide valuable insights into the neural and behavioral correlates of youth living in RC, longitudinal studies are necessary to evaluate how brain function and attachment-related symptoms evolve over time.

Despite these limitations, this study represents a significant step forward as the first assessment of brain function in youth from small-group family-like RC settings in Japan. Gaining the cooperation of child welfare agencies and RC facility administrators was a critical achievement, enabling novel insights into this population. Future studies should aim to build on this foundation by conducting longitudinal research that maintains sustained engagement with RC youth, allowing for a more comprehensive evaluation of their adaptive changes over time.

## 5 Conclusion

The current findings may reflect the impact of caregiving environments on attachment-related symptoms and functional brain networks. Youth raised in family-like RC homes exhibited higher levels of RAD and DSED symptoms than their peers raised in birth families. Interestingly, RAD symptoms were positively correlated with LLG-FMC connectivity, which was lower in the RC group than in the NRC group, but negatively correlated with the time spent in family-like RC homes. These findings suggest the possibility that stable, high-quality care environments may have a mitigating effect on the adverse developmental outcomes associated with early-life adversity. This suggests the importance of providing supportive caregiving settings to facilitate recovery and adaptive neurodevelopment in vulnerable populations.

## CRediT authorship contribution statement

**Shoko Shimada:** Writing – original draft, Visualization, Methodology, Investigation, Funding acquisition, Formal analysis, Data curation, Conceptualization. **Toshiki Iwabuchi:** Writing – original draft, Visualization, Methodology, Formal analysis. **Motofumi Sumiya:** Writing – original draft, Visualization, Methodology, Formal analysis. **Koji Shimada:** Writing – review & editing, Methodology, Investigation, Formal analysis, Conceptualization. **Shinichiro Takiguchi:** Writing – review & editing, Investigation, Conceptualization. **Kai Makita:** Writing – review & editing, Investigation, Conceptualization. **Akiko Yao:** Writing – review & editing, Investigation, Data curation. **Takashi X. Fujisawa:** Writing – review & editing, Investigation, Conceptualization. **Atsushi Senju:** Writing – review & editing, Supervision, Project administration. **Akemi Tomoda:** Writing – review & editing, Supervision, Project administration, Funding acquisition.

## Ethics and consent statement

This study was conducted in accordance with the World Medical Association Declaration of Helsinki and the ethical guidelines for medical and health research involving human subjects in Japan. The research protocol was approved by the Research Ethics Committee of the University of Fukui (approval number 20210004). Written informed consent was obtained from all participants and their legal guardians prior to participation in the study. Participant privacy and confidentiality were strictly maintained throughout the research process.

## Declaration of generative AI and AI-assisted technologies in the writing process

During the preparation of this work, the authors used Deepl, Deepl Write, Grammarly, and ChatGPT in order to improve language clarity. After using these tools/services, the authors reviewed and edited the content as needed and take full responsibility for the content of the published article.

## Funding

This work was supported by 10.13039/501100001691JSPS KAKENHI [grant numbers 19H00617, 20K13950, 22K18586]. The funders had no role in the study design, data collection, analysis, interpretation, manuscript preparation, or the decision to submit the manuscript for publication.

## Declaration of competing interest

The authors declare that they have no known competing financial interests or personal relationships that could have appeared to influence the work reported in this paper.

## Data Availability

The authors do not have permission to share data.
