# Supplementary material for: Functional connectivity of youth in family-like residential care in Japan: Impact of reactive attachment disorder and disinhibited social engagement disorder symptoms
Source: Neuroimage Rep. 2026 Jan 23;6(1):100323. doi: 10.1016/j.ynirp.2026.100323 (PMC12860606; doi:10.1016/j.ynirp.2026.100323)
Supplement: Multimedia component 1 [file mmc1.docx]

**Supplementary Information for “Functional Connectivity of Youth in Family-Like Residential Care in Japan: Impact of Reactive Attachment Disorder and Disinhibited Social Engagement Disorder Symptoms”**

**Supplementary Methods**

**Supplementary Results**

**Supplementary Table 1.** Results of linear regression analyses on RAD and DSED

**Supplementary Table 2.** Robustness and Sensitivity Diagnostics for the Within-RC Regression Model

**Supplementary Table 3.** Identified clusters for Sensitivity Analysis 1

**Supplementary Table 4.** Identified clusters for Sensitivity Analysis 2

**Supplementary Figure 1.** Results of Sensitivity Analysis 1

**Supplementary Figure 2.** Results of Sensitivity Analysis 2

**Supplementary Methods**

*Robustness checks for the post-hoc characterization of MVPA-derived clusters*

To examine the robustness of the group differences in resting-state functional connectivity (rsFC), we performed a jackknife (leave-one-out) analysis in which one participant from each group was removed at each iteration. In this analysis, we conducted a two-sample *t*-test and recomputed the effect size (Cohen’s *d*) at each iteration to check the stability of the group differences. Additionally, we performed a participant-level bootstrap analysis with 5,000 resamples to quantify the sampling variability of the effect size. In each bootstrap iteration, participants were resampled with replacement within each group while maintaining the original group sizes. For each resampled dataset, we recomputed the group difference in rsFC and calculated the effect size (Cohen’s *d*). The resulting bootstrap distribution was used to derive the 95% confidence interval (CI). These analyses were conducted using in-house MATLAB scripts.

*Influence diagnostics and robustness checks for multiple linear regression analysis*

To assess whether the reported association between RAD scores and rsFC was influenced by a few extreme observations, we performed standard influence diagnostics and robustness analyses. We calculated Cook’s distance, leverage values, and DFBETAs, and identified influential cases using conventional thresholds (Cook’s D > 4/n, leverage > 2p/n, |DFBETA| > 2/√n). The model was then re-estimated after excluding these observations (sensitivity analysis). To further confirm robustness, we fitted a robust regression using M-estimation with Huber weights in which predictors were standardized and OLS estimates were used as initial values. Finally, we inspected LOESS-smoothed scatterplots and partial-residual plots to check for potential non-linear patterns in the focal FC predictor.

**Supplementary Results**

*Robustness checks for the post-hoc characterization of MVPA-derived clusters*

For the six rsFC identified by the post-hoc analysis (i.e., right occipital pole [ROP]-left lingual gyrus [LLG], ROP-left orbital cortex [LFOC], ROP-left frontal pole [LFP], ROP-right frontal orbital cortex [RFOC], LLG-frontal medial cortex [FMC], and LLG-right precentral gyrus [RPG]), a jackknife procedure confirmed that the group difference remained significant across all iterations (*p* < 0.05), suggesting that the effect was not driven by any single participant. The ranges of Cohen’s *d* obtained from the iterations were as follows: ROP-LLG, -1.736 to -1.543; ROP-LFOC, 1.092 to 1.360; ROP-LFP, 0.656 to 0.904; ROP-RFOC, 1.104 to 1.340; LLG-FMC, -1.031 to -1.260; LLG-RPG, -1.045 to -0.828. Positive values indicate higher functional connectivity for the RC group compared to the NRC group, while negative values indicate the opposite pattern. Additionally, we performed a participant-level bootstrap analysis to assess the robustness of the group differences and calculate the 95% CI for the effect size. The proportions of bootstrapped *p*-values less than 0.05 were as follows: ROP-LLG, 100%; ROP-LFOC, 99.5%; ROP-LFP, 83.3%; ROP-RFOC, 99.6%; LLG-FMC, 98.8%; LLG-RPG, 94.3%. The 95% CIs of Cohen’s *d* for group comparisons in rsFC were as follows: ROP-LLG, -2.265 to -1.098; ROP-LFOC, 0.666 to 1.787; ROP-LFP, 0.261 to 1.301; ROP-RFOC, 0.660 to 1.781; LLG-FMC, -1.698 to -0.612; LLG-RPG, -1.530 to -0.433. These distributions of bootstrapped *p*-values and Cohen’s *d* indicated that the observed group differences in these rsFC values were stable and not influenced by a small number of participants.

*Influence diagnostics and robustness checks*

Three participants exhibited unusually high influence according to standard diagnostics—each exceeding at least one conventional threshold for leverage (h > 2p/n ≈ 0.57) or Cook’s distance (D > 4/n ≈ 0.14). These high-leverage observations were located at the upper end of the FC distribution and were removed in a sensitivity analysis to verify the stability of the results. After excluding these cases, the associations that were significant in the main model—namely those involving LLG-FMC, duration, and ROP-LFOC—remained directionally and materially consistent (e.g., LLG-FMC: β = 6.81 full sample vs. 5.34 sensitivity; SPC = 0.24; z-difference = 0.51; 95% CI overlap = TRUE; classification = stable). In contrast, several non-significant ROP-seed predictors showed greater sensitivity (SPC ≥ 0.6, including ROP-RFOC, which exhibited a sign reversal), suggesting that influential points primarily affected secondary covariates rather than the focal FC–RAD association. The M-estimation robust regression (Huber weighting, standardized predictors) yielded coefficients comparable in sign and magnitude to those from the OLS model (e.g., for LLG-FMC, β= 1.18 after standardization), indicating that the main effects were not driven by a few high-leverage observations. Visual inspection of LOESS and partial-residual plots did not reveal systematic non-linear trends. Collectively, these analyses demonstrate that the key within-RC associations—particularly between RAD symptom severity and fronto-parietal FC—are robust to influential observations and model specification, although several secondary ROP-seed predictors were more sensitive to leverage effects.

*Results of sensitivity analyses for rsfMRI data*

Two additional participants were excluded from Sensitivity Analysis 1 under the same criteria as the main analysis (i.e., participants with more than 20% of fMRI volumes detected as outliers). This analysis adopted a more stringent scrubbing threshold, a narrower smoothing kernel, and a stricter denoising process than did the main analysis. Similar to the main analysis, Sensitivity Analysis 1 identified the right occipital pole (ROP) and left lingual gyrus (LLG) (see Supplementary Figure 1), only when a more liberal threshold was applied (voxel-level threshold of *p* < 0.005 and an FDR-corrected cluster-level threshold of *p* < 0.05). Their peak coordinates were very close to the regions identified by the main analysis (Supplementary Table 3). Likewise, Sensitivity Analysis 2 confirmed that similar LLG and ROP clusters were identified when full-scale intelligence quotient (FSIQ) was excluded from the covariates (see Supplementary Figure 2 and Supplementary Table 4). Although a liberal threshold was used in this analysis (voxel-level threshold of *p* < 0.005 and an FDR-corrected cluster-level threshold of *p* < 0.05), this result suggests that our findings were not strongly affected by the inclusion of FSIQ as a covariate in the main analysis.

**Supplementary Table 1.** Results of linear regression analyses on RAD and DSED.

|  | RAD | DSED |
| --- | --- | --- |
| (Intercept) | -0.333 | 2.844 |
|  | (3.145) | (4.192) |
| age | 0.166 | 0.027 |
|  | (0.203) | (0.263) |
| duration | -0.286* | 0.268 |
|  | (0.116) | (0.145) |
| LLG-FMC | 6.810** | 2.664 |
|  | (1.903) | (2.492) |
| LLG-RPG | 2.011 | -0.326 |
|  | (2.616) | (4.314) |
| ROP-BLG | -12.014 | 12.719 |
|  | (5.996) | (6.658) |
| ROP-LFOC | 10.583* | -0.243 |
|  | (4.485) | (6.203) |
| ROP-LFP | -3.894 | 5.314 |
|  | (3.640) | (4.234) |
| ROP-RFOC | -5.648 | -6.261 |
|  | (5.254) | (7.180) |
| Num.Obs. | 28 | 28 |
| * *p* < 0.05, ** *p* < 0.01, *** *p* < 0.001  *Note:* LLG, left lingual gyrus; FMC, frontal medial cortex; RPG, right precentral gyrus; ROP, right occipital pole; BLG, bilateral lingual gyrus; LFOC, left frontal orbital cortex; LFP, left frontal pole; RFOC, right frontal orbital cortex. | | |

**Supplementary Table 2.** Robustness and Sensitivity Diagnostics for the Within-RC Regression Model.

| Term | β (Full) | 95% CI | β_std | β (Sensitivity) | SPC | Stability |
| --- | --- | --- | --- | --- | --- | --- |
| LLG-FMC | 6.81 | [3.08, 10.54] | 0.37 | 5.34 | 0.24 | stable |
| LLG-RPG | 2.01 | [-3.117, 7.139] | 0.09 | 2.06 | 0.03 | stable |
| ROP-LFOC | 10.58 | [1.792, 19.373] | 0.33 | 7.85 | 0.30 | moderate |
| duration | -0.29 | [-0.513, -0.058] | -0.35 | -0.21 | 0.29 | moderate |
| ROP-BLG | -12.01 | [-23.767, -0.261] | -0.40 | -4.22 | 0.96 | sensitive |
| ROP-LFP | -3.89 | [-11.027, 3.24] | -0.16 | -2.06 | 0.62 | sensitive |
| ROP-RFOC | -5.65 | [-15.946, 4.649] | -0.18 | 3.41 | 2.00 | sensitive |
| age | 0.17 | [-0.233, 0.565] | 0.15 | 0.35 | 0.72 | sensitive |

*Note*: This table summarizes the robustness and influence-diagnostic results for the within-RC multiple regression model. “β (Full)” and its 95% confidence interval reflect the HC0-adjusted estimates from the full dataset. “β (Sensitivity)” represents the corresponding estimates after removing influential observations identified by standard thresholds for Cook’s distance (4/n), leverage (2p/n), and DFBETAs (|2/√n|). Standardized coefficients (β_std) provide effect-size estimates. SPC denotes the stability-performance coefficient, and “Stability” classifies each predictor as *stable*, *moderate*, or *sensitive* based on effect-size changes, CI overlap, and sign consistency.

**Supplementary Table 3.** Identified clusters for Sensitivity Analysis 1.

|  | Brain region | Peak cluster coordinates (MNI) | Voxels per cluster |
| --- | --- | --- | --- |
| Cluster 1: | Occipital pole right | +18 -96 -12 | 211 |
| Cluster 2: | Lingual gyrus left | -6 -70 0 | 104 |
| Cluster 3: | Cingulate gyrus | -24 +34 +6 | 100 |
| Cluster 4: | Lateral occipital cortex | +42 -60 +16 | 80 |
| Cluster 5: | Frontal pole right | +18 +56 -18 | 80 |

*Note:* MNI, Montreal Neurological Institute; MVPA, multivariate pattern analysis.

**Supplementary Table 4.** Identified clusters for Sensitivity Analysis 2.

|  | Brain region | Peak cluster coordinates (MNI) | Voxels per cluster |
| --- | --- | --- | --- |
| Cluster 1: | Lingual gyrus left | -12 -72 0 | 360 |
| Cluster 2: | Occipital pole right | +18 -100 -16 | 179 |

*Note:* MNI, Montreal Neurological Institute; MVPA, multivariate pattern analysis.

**Supplementary Figure 1.** Results of Sensitivity Analysis 1. To confirm the robustness of the main MVPA findings, some preprocessing and denoising steps were modified. The spatial smoothing kernel was reduced from 8 mm to 6 mm. The motion exclusion threshold was set to framewise displacement ≥ 0.5, and participants with more than 20% of their fMRI volumes detected as outliers were excluded. This resulted in a final sample size of 59 participants. In addition to aCompCor and outliers, Friston 24 head-motion parameters were regressed out in the denoising process. As with the main results, this analysis identified the right occipital pole (peak coordinate, [18, -96, -12]; cluster size, 211) and left lingual gyrus (peak coordinate, [-6, -70, 0]; cluster size, 104). A slightly liberal threshold was used here (a voxel-level threshold of *p* < 0.001 and an FDR-corrected cluster-level threshold of *p* < 0.05). MVPA, multivariate pattern analysis; L, left; R, right.

**Supplementary Figure 2.** Results of Sensitivity Analysis 2. This analysis was performed to check whether or not excluding FSIQ from the covariates affected the main MVPA results. The left lingual gyrus (peak coordinate, [-12, -72, 0]; cluster size, 360) and right occipital pole (peak coordinate, [18, -100, -16]; cluster size, 179) were identified. This indicates that the main MVPA findings are robust. A slightly liberal threshold was used here (a voxel-level threshold of *p* < 0.001 and an FDR-corrected cluster-level threshold of *p* < 0.05).
